# A Valuable New Compound Metal

**Published:** 1880-05

**Authors:** 


*A Valuable New Compound Metal.*—Mr. Spence, an Englishman, has invented a compound metal which, if it possesses the properties claimed for it in the paper read a short time since before the British Society of Arts, promises to be brought largely into industrial uses as more applicable to many purposes than the metals now employed therein. This new metal is composed of the sulphides of iron, lead and zinc, which, when melted together in certain proportions, form a homogeneous mass. It is said to possess great tenacity, and not to be subject to oxidization either by the action of air, water or the alkalies. Even the most powerful acids act upon it very slowly, and only when reduced to a fine powder. The melting point of the metal is as low as 310, which gives it, we are told, a remarkable fitness for fine castings, especially such as are required for printing and stereotype purposes. In art work of a higher kind, as in statuettes and ornamental designs reproduced by molding, the superiority is claimed for it that it is susceptible of the highest polish, and that it will take any color, from the dark blue of steel to that of bronze, silver or gold. Its cost, moreover, being but about one-fourth the price of lead, and its perfect freedom from oxidization will render it, in the opinion of the inventor and of the Society of Arts, of peculiar value for water pipes and cisterns, and in the manufacture of acids. It will be known commercially as "Spence's metal."

